# Assessing the Potential Value and Mechanism of *Kaji-Ichigoside F1* on Arsenite-Induced Skin Cell Senescence

**DOI:** 10.1155/2022/9574473

**Published:** 2022-01-11

**Authors:** Qibing Zeng, Sufei Du, Yuyan Xu, Fan Yang, Liping Wu, Nanlan Wang, Shuling Zhang, Shaofeng Wei, Guoze Wang, Shuai Zhang, Hongguang Lu, Peng Luo

**Affiliations:** ^1^The Affiliated Hospital of Guizhou Medical University & Key Laboratory of Environmental Pollution Monitoring and Disease Control, Ministry of Education & School of Public Health, Guizhou Medical University, Guiyang 550025, China; ^2^Guizhou Provincial Engineering Research Center of Food Nutrition and Health, School of Public Health, Guizhou Medical University, Guiyang 550025, China; ^3^Department of Interventional Radiology, The Affiliated Cancer Hospital of Guizhou Medical University, Guiyang 550000, China

## Abstract

Chronic exposure to inorganic arsenic is a major environmental public health issue worldwide affecting more than 220 million of people. Previous studies have shown the correlation between arsenic poisoning and cellular senescence; however, knowledge regarding the mechanism and effective prevention measures has not been fully studied. First, the associations among the ERK/CEBPB signaling pathway, oxidative stress, and arsenic-induced skin cell senescence were confirmed using the HaCaT cell model. In the arsenic-exposed group, the relative mRNA and protein expressions of ERK/CEBPB signaling pathway indicators (ERK1, ERK2, and CEBPB), cell cycle-related genes (*p21*, *p16^INK4a^)*, and the secretion of SASP (IL-1*α*, IL-6, IL-8, TGF-*β*1, MMP-1, MMP-3, EGF, and VEGF) and the lipid peroxidation product (MDA) were significantly increased in cells (*P* < 0.05), while the activity of antioxidant enzyme (SOD, GSH-Px, and CAT) was significantly decreased (*P* < 0.05), and an increased number of cells accumulated in the G1 phase (*P* < 0.05). Further *Kaji-ichigoside F1* intervention experiments showed that compared to that in the arsenic-exposed group, the expression level of the activity of antioxidant enzyme was significantly increased in the *Kaji-ichigoside F1* intervention group (*P* < 0.05), but the indicators of ERK/CEBPB signaling pathway, cell cycle-related genes, and SASP were significantly decreased (*P* < 0.05), and the cell cycle arrest relieved to a certain extent (*P* < 0.05). Our study provides some limited evidence that the ERK/CEBPB signaling pathway is involved in low-dose arsenic-induced skin cell senescence, through regulating oxidative stress. The second major finding was that *Kaji-ichigoside F1* can downregulate the ERK/CEBPB signaling pathway and regulate the balance between oxidation and antioxidation, alleviating arsenic-induced skin cell senescence. This study provides experimental evidence for further understanding of *Kaji-ichigoside F1*, a natural medicinal plant that may be more effective in preventing and controlling arsenic poisoning.

## 1. Introduction

Arsenic contamination of groundwater, air, or food is a major environmental public health issue worldwide affecting more than 220 million of people [1, 2]. Skin lesions are the hallmark signs of chronic arsenic exposure; the basic pathological changes include skin hyperpigmentation and hyperkeratosis, Bowen's disease, basal cell carcinoma, and squamous cell carcinoma [3]. As a ubiquitous biological phenomenon, cellular senescence is characterized by irreversible cell cycle arrest and senescence-associated secretory phenotype (SASP) [4, 5]. It is the main cause of many chronic noncommunicable diseases and can increase the susceptibility of diseases [4, 6–8]. For chronic arsenic poisoning, a little of studies show that arsenic can increase the senescence-related *β*-galactosidase (SA-*β*-gal) activity, upregulate the expression of *p53*, *p21*, and *p16^INK4a^*, and promote the secretion of SASP [9–11]. Although previous studies show the relationship between arsenic poisoning and cellular senescence, there is very little known about the mechanism and effective preventive measures, which has made recent progress in the control of endemic arsenic poisoning slow.

CCAAT-enhancer-binding protein *β* (CEBPB), also known as nuclear factor interleukin-6, is an important member of the CCAAT-enhancer-binding protein family of transcription factors [12]. Previous study shows that cell cycle arrest of senescent cells is regulated by the *p53*/*p21* and *p16^INK4a^/RB* tumor suppressor pathway, while SASP is regulated by enhancer remodeling and transcription factor activation [4], such as CEBPB and mitogen-activated protein kinase (MAPK) signal pathway. Extracellular-regulated protein kinases (ERK) are one of the members of MAPK, including two members, ERK1 and ERK2, which mainly mediate cell proliferation, migration, differentiation, and apoptosis [13]. In cellular senescence, limited studies [14–16] show that ERK phosphorylation can regulate the expression of downstream transcription factor CEBPB, and the activation of CEBPB can break the balance between oxidation and antioxidation, thereby inducing oxidative stress [17, 18]. Furthermore, the activation of CEBPB can coordinate with nuclear factor *κ*B to regulate the secretion of multiple SASP components [19], including inflammatory factors (interleukin-1*α* (IL-1*α*), interleukin-6 (IL-6), interleukin-8 (IL-8), and transforming growth factor *β* (TGF-*β*)), matrix remodeling factors (matrix metallopeptidase-1 (MMP-1), matrix metallopeptidase-3 (MMP-3)), and growth factors (epidermal growth factor (EGF), vascular endothelial growth factor (VEGF)). The above studies suggest that the ERK/CEBPB signaling pathway can participate in the occurrence of cell senescence by regulating SASP. Although there is currently no evidence that arsenic can participate in the occurrence of skin cell senescence through the ERK/CEBPB signaling pathway, arsenic can activate the ERK signaling pathway which has been accepted by several studies [20–22]. And previous studies [9–11] show that arsenic can induce skin cell senescence. It is speculated that arsenic may regulate the secretion of SASP by activating the ERK/CEBPB signaling pathway and participate in the pathogenic process of arsenic-induced skin cell senescence.

In recent years, the global economic burden of chronic diseases caused by cellular senescence has continued to rise, and the research and development of effective prevention and treatment measures for aging have become the focus and challenge of the world [6]. In the field of arsenic poisoning, lifestyle changes [2] and preventive medicines (such as vitamins [23, 24], trace elements [25], and natural medicinal plant [26–28]) have gradually been accepted by scholars worldwide. However, there are very limited effective targeted prevention and treatment measures for skin cell senescence caused by arsenic. *Rosa roxburghii* is a natural medicinal and edible plant that is unique to the mountainous area of southwest China and has antioxidant and antihypoxia effects [29, 30]. Its main active ingredients include polysaccharides, flavonoids, superoxide dismutase, vitamin C, and triterpenes. Oxidative stress deepens the understanding of the cellular senescence process and provides promising tools for the development of new therapeutic strategies [31]. Our previous animal study found that *Rosa roxburghii* can attenuate arsenic poisoning by regulating element balance and oxidative stress [27]. However, we would like to know which active ingredients in *Rosa roxburghii* are involved in the above process. *Kaji-ichigoside F1* is a triterpenoid compound extracted from *Rosa roxburghii* which accounts for the largest proportion of triterpenoid active ingredients. Since *Rosa roxburghii* has achieved some positive effects in the prevention and treatment of arsenic poisoning and antioxidation process, it is of great importance to explore whether *Kaji-ichigoside F1* (the main active ingredient of *Rosa roxburghii*) can improve the skin cell senescence caused by arsenic and its possible mechanism. Furthermore, this study is of great important scientific significance and application value to better explain the role of *Rosa roxburghii* in arsenic poisoning.

In this study, by observing the level changes of oxidative stress (malondialdehyde (MDA), superoxide dismutase (SOD), glutathione peroxidase (GSH-Px), and catalase (CAT)) and SASP (IL-1*α*, IL-6, IL-8, TGF-*β*1, MMP-1, MMP-3, EGF, and VEGF) and the expression changes in mRNA and protein of the indicators related to the ERK/CEBPB signaling pathway (ERK1, ERK2, and CEBPB) and cell cycle and related genes (*p21*, *p16^INK4a^*) in cells or cell culture supernatant, the goal was to study the possible mechanism of arsenic-induced skin cell senescence. A *Kaji-ichigoside F1* intervention study was then conducted to observe the differences in the above biomarkers in the cells or cell culture supernatant of the various groups to explore the possible application value of *Kaji-ichigoside F1* in arsenic-induced skin cell senescence. This study will help to have a better understanding of the mechanisms of *Kaji-ichigoside F1* in skin cell senescence caused by exposure to arsenic, and our results will identify a possible natural medicinal plant that can be used to screen the effective prevention and control strategies.

## 2. Materials and Methods

### 2.1. Cell Culture and Treatments

In this study, human immortalized keratinocytes (HaCaT) were used to construct an arsenic exposure model and a *Kaji-ichigoside F1* intervention cell model; the source of the cell line and the culture method were performed as those described previously [32]. Sodium arsenite and *Kaji-ichigoside F1* were purchased from Merck (Germany) and PUSH Biotechnology (China). According to the design of this study, HaCaT cells were treated with 0.00, 0.05, 0.10, and 0.25 *μ*M NaAsO_2_ for 24, 48, and 72 hours (h). *Kaji-ichigoside F1* concentration (10^−15^~10^−9^ M) without cytotoxicity was used to treat arsenic-exposed HaCaT cells. The CCK8 method was used to detect the effect of *Kaji-ichigoside F1* on the viability of arsenic-exposed HaCaT cells. Based on the cell viability test results, the concentration of *Kaji-ichigoside F1* (10^−12^M) that can significantly increase the viability of arsenic-exposed HaCaT cells was selected as the intervention dose. After the HaCaT cells were treated with 0.25 *μ*M NaAsO_2_ for 72 h, they were replaced with fresh medium and then treated with *Kaji-ichigoside F1* (10^−12^M) for 24 h. The intervention experiment included the control group, NaAsO_2_ (0.25 *μ*M) treatment group, *Kaji-ichigoside F1* (10^−12^M) group, and *Kaji-ichigoside F1* (10^−12^M) combined with NaAsO_2_ (0.25 *μ*M) intervention group. A total of 6 repeated experiments and 3 replicates in each group were used in this study.

### 2.2. Determination of Antioxidant Enzyme Activity and Lipid Peroxidation Product

The SOD, GSH-Px, CAT, and MDA were determined using kits from Nanjing Jiancheng Bioengineering (China). In accordance with a previous study [33], the thiobarbituric acid method and dithiodinitrobenzoic acid method were used to measure the level of MDA and SOD. And the levels of CAT and GSH-Px were determined by ammonium molybdate method and colorimetric method. And the levels of SOD, GSH-Px, CAT, and GSH-Px were normalized by protein concentrations.

### 2.3. Enzyme-Linked Immunosorbent Assays

Human-specific IL-1*α*, IL-6, IL-8, TGF-*β*1, MMP-1, MMP-3, EGF, and VEGF in cell supernatant were detected using the kits from CUSABIO Technology LLC (China). And the method of above indicators was performed as the previous study [32]. A parallel sample design was used in this study, and each sample was repeated 3 times. Finally, the average absorbance was calculated for evaluating the concentration of SASP.

### 2.4. Quantitative Real-Time PCR

According to the manufacturer's instructions, after the total RNA isolation, quantitative real-time PCR method was used to determine the levels of ERK1, ERK2, CEBPB, *p21*, and *p16^INK4a^* mRNA expression by the CFX96 Touch Deep Well Real-Time PCR (Bio-Rad, USA), which is the same as those described previously [32]. Table S1 shows the primer sequences of all genes which were synthesized by Takara Biomedical Technology Co., Ltd. (China). GAPDH is used as an internal control to evaluate the relative expression of various genes.

### 2.5. Western Blot Analysis

As those described in the previous study [32], the same method was used to extract total protein for protein quantification and subsequent western blot analysis. In short, the immune complex in PVDF membrane (Millipore, USA) was measured by using a chemiluminescence imaging system of Bio-Rad (ChemiDoc, USA). The GAPDH was used as an internal control to evaluate the relative expression of ERK1, ERK2, *p-*ERK, CEBPB, *p21*, and *p16^INK4a^* and to normalize the blot. The above antibodies were obtained from Abcam (USA).

### 2.6. Flow Cytometry Assay

According to the manufacturer's instructions, the cell cycle was measured using the propidium iodide cell cycle detection kit from KeyGEN Biotech, China, the same as those described previously [34]. The percentages of different cell cycles (G1, G2, and S) were determined using flow cytometry (FACSCelesta, BD, USA).

### 2.7. Statistical Analysis

All data were entered and analyzed using Excel 2019 and SPSS 22.0 software. After the data has been tested by normal distribution, one-way analysis of variance (ANOVA) was used to compare the differences between the overall groups, and the least significant difference (LSD) test was used for further subgroup analysis. The mean and standard deviation were used to describe the distribution characteristics of the data of 6 independent experiments. When the *P* value was less than 0.05, the difference was statistically significant.

## 3. Results

### 3.1. Arsenic Can Induce the Skin Cell Senescence by Promoting the Level of SASP Secretion and Cell Cycle Arrest in Cells

**Figure 1:**
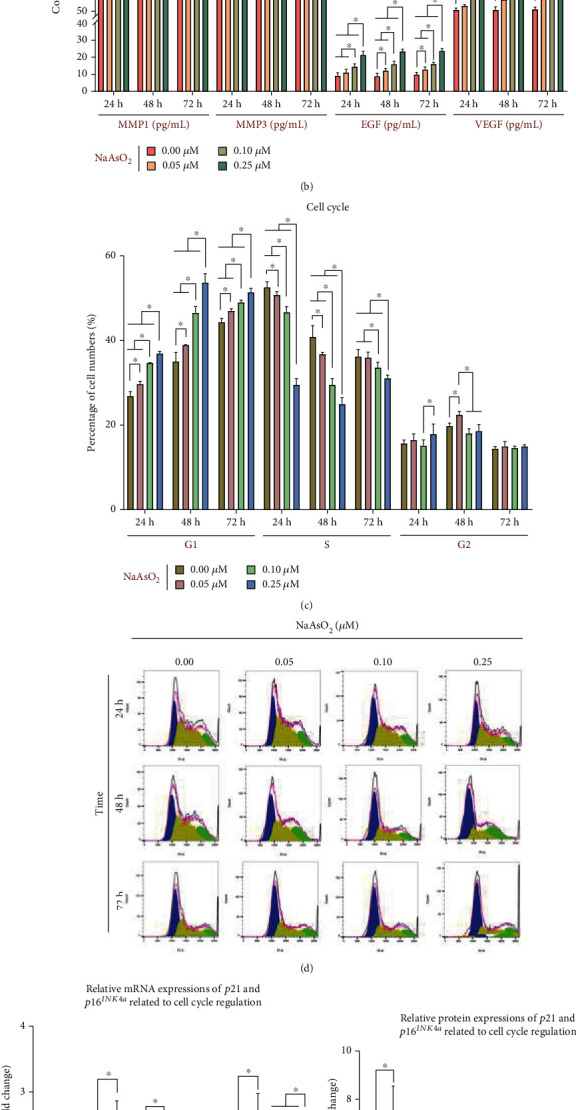
Arsenic can induce the skin cell senescence by promoting the level of SASP secretion and cell cycle arrest in cells. The *in vitro* results were based on 6 independent experiments. All data are presented as mean ± standard deviation. ^∗^*P* < 0.05. (a) The concentration of inflammatory factors. (b) The concentration of matrix remodeling factors and growth factors. (c) The cell cycle for each group. (d) The cell cycle analysis of flow cytometry. (e) The relative mRNA expression of *p21* and *p16^INK4a^* to cell cycle regulation. (f) The relative protein expression of *p21* and *p16^INK4a^* to cell cycle regulation. (g) The western blot for *p21* and *p16^INK4a^*.

To assess the effect of NaAsO_2_ on cellular senescence, 0.00, 0.05, 0.10, and 0.25 *μ*M NaAsO_2_ were used to treatment HaCaT cells for 24, 48, or 72 h. Figures 1(a) and 1(b) show the secretion of SASP, including inflammatory factors, matrix remodeling factors, and growth factors. From the graph above, we can see that compared with the control group, the concentration of IL-1*α*, IL-6, IL-8, MMP-1, MMP-3, EGF, and VEGF in the 0.05, 0.10, and 0.25 *μ*M NaAsO_2_-exposed groups was increased (*P* < 0.05), except MMP1 and EGF in the 0.05 *μ*M NaAsO_2_-exposed group for 24 h (*P* > 0.05); and the dose- and time-dependent increase was more obvious at 0.25 *μ*M NaAsO_2_ treatment 72 h (*P* < 0.05). Additionally, the concentration of TGF-*β*1 in the 0.10 *μ*M (48 and 72 h) and 0.25 *μ*M (24, 48, and 72 h) NaAsO_2_-exposed groups was increased than that in the control group and 0.05 *μ*M NaAsO_2_-exposed group (*P* < 0.05), except TGF-*β*1 in the 0.10 *μ*M NaAsO_2_ treatment for 24 h. Compared with the 0.10 *μ*M NaAsO_2_-exposed group, the concentration of TGF-*β*1 in the 0.25 *μ*M (48 and 72 h) NaAsO_2_-exposed groups was increased (*P* < 0.05). The concentration of the SASP among the other groups did not change significantly (*P* > 0.05).

We sought to determine if arsenic induced cell cycle arrest within different periods over a range of doses. After the HaCaT cells were exposed to 0.05~0.25 *μ*M NaAsO_2_, the cell numbers of G1 phase are higher than those observed in the control group at 24 h, 48 h, and 72 h (*P* < 0.05), and the dose-dependent increase was more obvious at 72 h (*P* < 0.05) (Figures 1(c) and 1(d)). However, compared with the control group, the cell numbers of S phase were gradually decreased in various NaAsO_2_-exposed groups (*P* < 0.05) (Figures 1(c) and 1(d)). Additionally, the cell numbers of G2 phase in the 0.10 *μ*M NaAsO_2_-exposed group are higher than those observed in the control group at 48 h (*P* < 0.05) (Figures 1(c) and 1(d)). The cell numbers of G2 phase did not change significantly among the other NaAsO_2_-exposed groups compared with the control group (*P* > 0.05).

Figures 1(e)–1(g) show the relative mRNA and protein expressions of *p21* and *p16^INK4a^* related to cell cycle regulation in various groups. From the graph above, we can see that the relative expression of *p21* and *p16^INK4a^* in the 0.05, 0.10, and 0.25 *μ*M NaAsO_2_-exposed groups is higher than that in the control group (*P* < 0.05), except that of *p21* in the 0.05 *μ*M NaAsO_2_-exposed group (72 h) and that of *p16^INK4a^* in the 0.05 *μ*M NaAsO_2_-exposed group (48 h) (*P* > 0.05); and the dose- and time-dependent increase was more obvious at 0.25 *μ*M NaAsO_2_-exposed treatment 72 h (*P* < 0.05). Although the changes of the relative protein expressions of *p21* and *p16^INK4a^* are not as obvious as mRNA expressions, compared with the control group, the relative protein expressions of *p21* in the 0.05, 0.10, and 0.25 *μ*M NaAsO_2_-exposed groups are higher than those in the control group (*P* < 0.05), except those of *p21* in the 0.05 *μ*M NaAsO_2_ treatment group (24 and 72 h) (*P* > 0.05). Additionally, the relative protein expressions of *p16^INK4a^* in the 0.10 *μ*M (72 h) and 0.25 *μ*M (48 and 72 h) NaAsO_2_-exposed groups were increased compared to those observed in the control group (*P* < 0.05). The expression of *p21* and *p16^INK4a^* did not change significantly among the other NaAsO_2_-exposed groups compared with the control group (*P* > 0.05).

### 3.2. ERK/CEBPB Signaling Pathway Participates in Arsenic-Induced Skin Cell Senescence through Regulating Oxidative Stress

**Figure 2:**
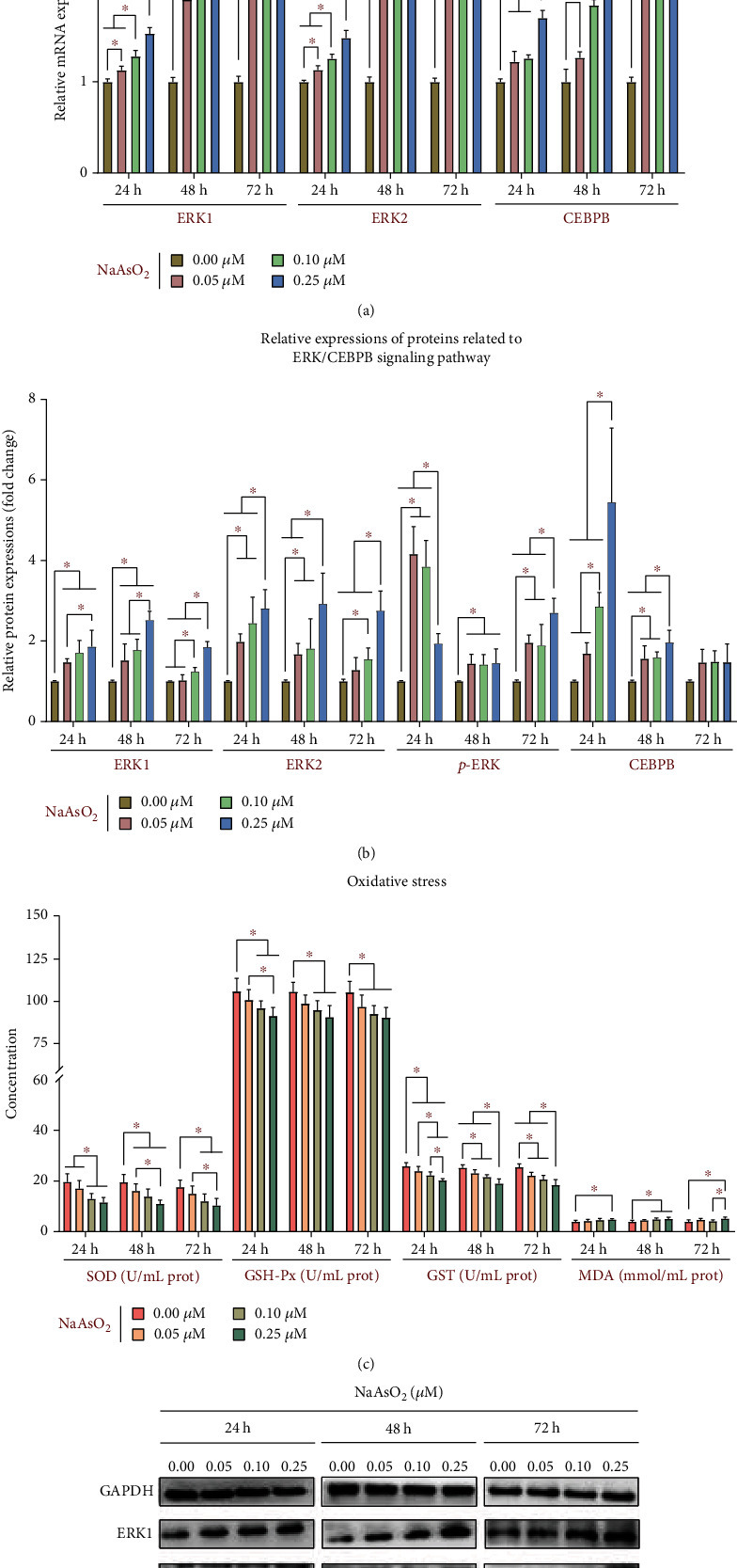
The ERK/CEBPB signaling pathway participates in arsenic-induced skin cell senescence through regulating oxidative stress. The *in vitro* results were based on 6 independent experiments. All data are presented as mean ± standard deviation. ^∗^*P* < 0.05. (a) The relative mRNA expression of genes related to the ERK/CEBPB signaling pathway. (b) The relative protein expression related to the ERK/CEBPB signaling pathway. (c) The concentration of oxidative stress indicators. (d) The western blot for the indicators of the ERK/CEBPB signaling pathway.

To understand whether the ERK/CEBPB signaling pathway participates in arsenic-induced skin cell senescence, the relative mRNA and protein expressions of ERK1, ERK2, and CEBPB in cells were determined. Figures 2(a), 2(b), and 2(d) show that the relative mRNA expression of ERK1, ERK2, and CEBPB in the 0.05, 0.10, and 0.25 *μ*M NaAsO_2_-exposed groups was increased than that in the control group (*P* < 0.05), and the dose- and time-dependent increase was more obvious at 0.25 *μ*M NaAsO_2_-exposed group 72 h (*P* < 0.05). Compared with the control group, the relative protein expression of ERK1, ERK2, *p-*ERK, and CEBPB in the 0.05, 0.10, and 0.25 *μ*M NaAsO_2_-exposed groups is higher than that in the control group (*P* < 0.05), except that of ERK2 in the 0.05 *μ*M NaAsO_2_ treatment group (72 h) (*P* > 0.05), that of CEBPB in the 0.05 *μ*M NaAsO_2_-exposed group (24 h) (*P* > 0.05), and that of CEBPB in various NaAsO_2_ treatment group (72 h) (*P* > 0.05). The relative expression of ERK1, ERK2, and CEBPB did not change significantly among the other NaAsO_2_-exposed groups compared with the control group (*P* > 0.05).

Subsequently, we validated the relationship between oxidative stress and cellular senescence. As illustrated in Figure 2(c), the antioxidant enzyme activity of CAT in the 0.05, 0.10, and 0.25 *μ*M NaAsO_2_-exposed groups is lower than that in the control group (*P* < 0.05). Compared with the control group, the concentration of SOD and GSH-Px in the 0.10 and 0.25 *μ*M NaAsO_2_ treatment group, that of SOD in the 0.05 *μ*M NaAsO_2_ treatment group (48 h), and that of GSH-Px in the 0.05 *μ*M NaAsO_2_ treatment group (72 h) were decreased (*P* < 0.05). Additionally, the lipid peroxidation product MDA in the 0.25 *μ*M NaAsO_2_ treatment group is higher than that in the control group (*P* < 0.05), and the MDA in the 0.10 *μ*M NaAsO_2_ treatment group was increased compared to those observed in the control group (*P* < 0.05). The SOD, GSH-Px, CAT, and MDA did not change significantly among the other NaAsO_2_ groups compared with the control group (*P* > 0.05).

### 3.3. Kaji-Ichigoside F1 Can Downregulate the ERK/CEBPB Signaling Pathway and Regulate the Balance between Oxidation and Antioxidation, Alleviating Arsenic-Induced Skin Cell Senescence

**Figure 3:**
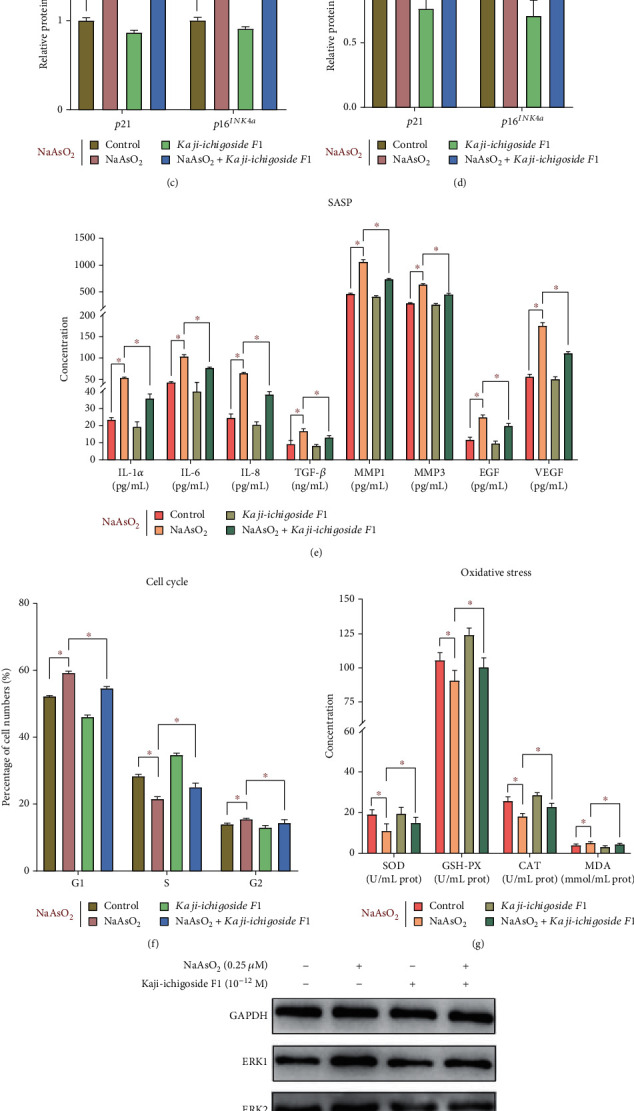
*Kaji-ichigoside F1* can downregulate the ERK/CEBPB signaling pathway and regulate the balance between oxidation and antioxidation, alleviating arsenic-induced skin cell senescence. The *in vitro* results were based on 6 independent experiments. All data are presented as mean ± standard deviation. ^∗^*P* < 0.05. (a) The relative mRNA expression of genes related to the ERK/CEBPB signaling pathway. (b) The relative protein expression related to the ERK/CEBPB signaling pathway. (c) The relative mRNA expression of *p21* and *p16^INK4a^* to cell cycle regulation. (d) The relative protein expression of *p21* and *p16^INK4a^* to cell cycle regulation. (e) The concentration of SASP. (f) The cell cycle for each group. (g) The concentration of oxidative stress indicators. (h) The western blot for the indicators of the ERK/CEBPB signaling pathway. (i) The western blot for *p21* and *p16^INK4a^*. (j) The cell cycle analysis of flow cytometry.

To assess the potential value of *Kaji-ichigoside F1* on arsenic-induced skin cell senescence, the changes of the indicators related to the ERK/CEBPB signaling pathway, cell cycle and related genes, oxidative stress, and SASP in cells or cell culture supernatant were observed after treatment with *Kaji-ichigoside F1*. Figures 3(a), 3(b), and 3(h) show that the relative mRNA and protein expression of ERK1, ERK2, and CEBPB in the NaAsO_2_+*Kaji-ichigoside F1* treatment group is lower compared to that observed in the NaAsO_2_ treatment group (*P* < 0.05). Meanwhile, the concentration of SOD, GSH-Px, and CAT in the NaAsO_2_+*Kaji-ichigoside F1* treatment group is higher than that in the NaAsO_2_ group (Figure 3(g)) (*P* < 0.05). As revealed in Figures 3(c), 3(d), and 3(i), *Kaji-ichigoside F1* reverses the outcome for *p21* and *p16^INK4a^* caused by arsenic. Compared with that in the NaAsO_2_ treatment group, the relative mRNA and protein expression of *p21* and *p16^INK4a^* in the NaAsO_2_+*Kaji-ichigoside F1* treatment group was decreased (*P* < 0.05). Subsequently, we determined the cell cycle after treatment with *Kaji-ichigoside F1*. The data shown in Figures 3(f) and 3(j) indicate that the effect of arsenic on G1 phase cell cycle arrest was restored by treatment with *Kaji-ichigoside F1*. The cell numbers of G1 and G2 phase in the NaAsO_2_+*Kaji-ichigoside F1* treatment group are lower than those in the NaAsO_2_ group (Figure 3(g)) (*P* < 0.05). In contrast, compared with those in the NaAsO_2_ group, cell numbers of S phase in the NaAsO_2_+*Kaji-ichigoside F1* treatment group were increased (*P* < 0.05). Additionally, the above positive regulation of *Kaji-ichigoside F1* reduces the secretion of SASP. As indicated in Figure 3(e), the concentration of IL-1*α*, IL-6, IL-8, TGF-*β*1, MMP-1, MMP-3, EGF, and VEGF in the NaAsO_2_+*Kaji-ichigoside F1* treatment group is lower than that in the NaAsO_2_ group (*P* < 0.05).

## 4. Discussion

Endemic arsenic poisoning, as a biogeochemical disease that seriously endangers human health, has become an important global public health problem [1, 2]. Guizhou Province is a unique area of arsenic poisoning worldwide, caused by exposure to great amounts of arsenic-contaminated air and food, from the burning of coal in unventilated indoor stoves [2]. In the past 60 years, under the correct leadership of the Chinese government and the unremitting efforts of the vast number of endemic disease prevention and control scholars, the prevention and treatment of arsenic poisoning in Guizhou Province have achieved world-renowned achievements, in blocking high arsenic exposure and health education [35]. However, the health hazards of endemic arsenic poisoning are cumulative and irreversible. In addition, the pathogenic mechanism of endemic arsenic poisoning is not yet clear and there are very limited effective therapeutic drugs. These factors have become the bottleneck restricting the continuous control and elimination of the endemic arsenic poisoning.

Chronic arsenic exposure can cause damage to multiple organs and multiple systems [36–38], but the response to arsenic exposure usually first appears in the skin [39]. Furthermore, our study focused on the skin, one of the target organs of arsenic. Cellular senescence is a ubiquitous biological phenomenon characterized by irreversible cell cycle arrest and senescence-associated secretory phenotype, accompanied by increased expression of senescence-related genes and proteins [4, 5]. Previous studies [9–11] have noted the importance of cellular senescence in arsenic poisoning. It has been found that 1~5 *μ*M arsenic trioxide can increase the activity of SA-*β*-gal and the relative protein expression of *p16^INK4a^*, *p53*, and *p21*, inducing senescence in human articular chondrocytes [10]. In bone marrow mesenchymal stem cells, 1 *μ*M arsenic trioxide treatment for 48 h has been found to enhance SA-*β*-gal activity and *p21* protein expression in the cells [40]. Another study has also shown that 10 ppm arsenic acid increased the SA-*β*-gal activity and induced the mouse skin fibroblast cell arrest in G2/M phase [41]. Furthermore, after 50 ppm arsenic exposure for 12 months, the expression of *p16*^INK4a^ in mice increased significantly [11]. In particular, a population-based evidence from India has revealed that the number of senescent cells and the expression of *p53* and *p21* in arsenic-exposed individuals increased significantly [9]. Given that high arsenic exposure in Guizhou, China, has been well controlled, in this study, we first sought to determine whether low dose of arsenic induced cellular senescence in the skin. By observing the level changes of SASP, cell cycle, and related genes, the results demonstrated that the concentration of SASP (including IL-1*α*, IL-6, IL-8, TGF-*β*1, MMP-1, MMP-3, EGF, and VEGF) and the relative expression of cell cycle-related genes (*p21* and *p16^INK4a^*) in the arsenic exposure group gradually increased. Additionally, sodium arsenite reduced the S phase cell numbers, and an increased number of cells accumulated in the G1 phase. These findings suggested that arsenic can induce cell cycle arrest by upregulating the expression levels of cell cycle regulation-related genes and promote the secretion of SASP. Our experiments provide some limited evidence supporting our hypothesis that low dose of arsenic can induce HaCaT cell senescence. In addition, this study also suggests that in low-dose arsenic-exposed areas in the skin damage health monitoring of the population, cellular senescence needs more attention.

The ERK signaling pathway has been found to be involved in the cellular senescence [42, 43]. Further studies [14–16] have found that the regulation of cell senescence by ERK is achieved by activating the transcription factor CEBPB. On the one hand, the activation of CEBPB can break the balance between oxidation and antioxidation [17, 18]; and on the other hand, CEBPB can also regulate the secretion of SASP [4, 19]. Although there is currently no evidence that arsenic can participate in the occurrence of skin cell senescence through the ERK/CEBPB signaling pathway, arsenic can activate the ERK signaling pathway which has been accepted by several studies [20–22]. In this study, the results indicated that arsenic could increase the relative mRNA and protein expressions of ERK1 and ERK2 and that there is a significant dose and time-effect relationship among arsenic and the above indicators. Additionally, the study also found that arsenic can increase the relative mRNA and protein expressions of CEBPB. Previous studies have found that ERK regulates the transcription factor CEBPB through phosphorylation. Our results provide further support for the above hypothesis. In this study, the results showed that the relative mRNA and protein expressions of *p-*ERK in the various arsenic-exposed groups are higher than those in the control group and that there is a significant dose and time-effect relationship between arsenic and *p-*ERK. Combined with the results that low dose of arsenic can induce HaCaT cell senescence, this finding provides evidence that the ERK/CEBPB signaling pathway is involved in low-dose arsenic-induced skin cell senescence.

Oxidative stress is one of the main mechanisms of skin lesions caused by arsenic, which has been accepted by more and more scholars [39]. Ample published evidence suggests that low dose of reactive oxygen species can stimulate the body protective response and delay the cell senescence process in the early stage [44]; when the cell senescence-related oxidative damage was cumulated, these reactive oxygen will exacerbate cell senescence-related oxidative damage and accelerate the cell senescence process [31]. A recent study provided conclusive evidence for the first time that oxidative stress directly acts on telomeres to accelerate cell senescence [45]. In this study, we investigated NaAsO_2_-induced oxidative stress status in HaCaT cells. The results indicated that NaAsO_2_ exposure in HaCaT cells significantly increased the MDA levels. Conversely, the antioxidant enzymes SOD, GSH-Px, and CAT decreased, and that there is a significant dose and time-effect relationship among arsenic and the activity of antioxidant enzymes. The free radical theory proposes that cellular damage caused by excessive oxidative stress greatly promotes the development of the cell senescence phenotypes [46]. Our results also support the hypothesis of free radical theory that arsenic can promote the secretion of SASP through oxidative stress. The results of this study confirm the results of previous research, and previous research has demonstrated that ERK phosphorylation can regulate the expression of downstream transcription factor CEBPB, and the activation of CEBPB can break the balance between oxidation and antioxidation, thereby inducing oxidative stress. In accordance with the above present results, it suggests that the ERK/CEBPB signaling pathway is involved in low-dose arsenic-induced secretion of SASP, through regulating oxidative stress. Furthermore, several studies [47–49] have found that the high level of oxidative stress can exacerbate cell cycle arrest. And cell cycle arrest and SASP secretion are the characteristics of cell senescence. Therefore, this finding provides limited evidence that the ERK/CEBPB signaling pathway is involved in low-dose arsenic-induced skin cell senescence, through regulating oxidative stress. This study provides an experimental basis for further understanding of the mechanisms of arsenic-induced skin cell senescence.

Oxidative stress deepens the understanding of the cellular senescence process and provides promising tools for the development of new therapeutic strategies [31]. *Rosa roxburghii* is a natural medicinal and edible plant that is unique to the mountainous area of southwest China, in which its main active ingredients are flavonoid of *Rosa roxburghii Tratt* and triterpenoids of *Rosa roxburghii Tratt*, and has antioxidant and antihypoxia effects [29, 30]. Recent study has shown that *Rosa roxburghii* can attenuate arsenic poisoning by regulating element balance and oxidative stress [27]. In this study, we sought to determine whether the *Kaji-ichigoside F1* (the main active ingredient of *Rosa roxburghii*) can improve the skin cell senescence caused by arsenic and its possible mechanism. The results indicated that *Kaji-ichigoside F1* can enhance the activity of antioxidant enzyme (SOD, GSH-Px, and CAT) and decrease the content of lipid peroxidation product (MDA). Additionally, *Kaji-ichigoside F1* reduces the secretion of SASP in the arsenic-exposed HaCaT cells, including IL-1*α*, IL-6, IL-8, TGF-*β*1, MMP-1, MMP-3, EGF, and VEGF. And the cell cycle arrest relieved to a certain extent. Based on the present results, this study indicated that *Kaji-ichigoside F1* can improve the skin cell senescence by decreasing the oxidative stress. One interesting finding is that *Kaji-ichigoside F1* can decrease the relative mRNA and protein expressions of ERK1, ERK2, CEBPB, *p21*, and *p16^INK4a^* in the arsenic-exposed HaCaT cells. This finding provides more evidence to support the mechanism hypothesis of arsenic-induced skin cell senescence; the hypothesis found that the ERK/CEBPB signaling pathway is involved in low-dose arsenic-induced skin cell senescence, through regulating oxidative stress. A puzzling result is that *Kaji-ichigoside F1* alone can increase the expression level of ERK protein, but after treatment with *Kaji-ichigoside F1* interfering in arsenic-exposed HaCaT cells, the expression level of ERK protein is significantly reduced. Does this mean that *Kaji-ichigoside F1* interacts with sodium arsenite and affects the expression of ERK protein together? It suggests that the interaction of *Kaji-ichigoside F1* and arsenic on ERK expression should be further explored in the future to reveal the special effects of *Kaji-ichigoside F1* on arsenic-exposed cells. Furthermore, the results of this study also deepen our understanding of the intervention mechanism of *Kaji-ichigoside F1* in skin cell senescence caused by arsenic, namely, *Kaji-ichigoside F1* can downregulate the ERK/CEBPB signaling pathway and regulate the balance between oxidation and antioxidation, alleviating arsenic-induced skin cell senescence. This finding represents an important issue for future research; the animal experiments and population intervention studies can be used to confirm the application value of *Kaji-ichigoside F1*.

Since there has been no ideal animal model, the current study on arsenic-induced skin cell senescence mainly focuses on culturing keratinocytes in vitro. In this study, the results demonstrated that *Kaji-ichigoside F1* can downregulate the ERK/CEBPB signaling pathway and regulate the balance between oxidation and antioxidation, alleviating arsenic-induced skin cell senescence. This fits well with the drug development strategy of “conventional drug in new use” that more and more scholars have been paying attention to in recent years, as a drug development strategy, and many new indication drugs have been derived. This research has important scientific significance and practical application value for promoting the transformation and application of *Kaji-ichigoside F1* (as a traditional natural medicinal plant). Our research also has limitations. In view of the difficulty of obtaining skin samples from the population, the findings of this study are mainly based on changes in the expression of biomarkers reflecting arsenic-induced skin cell senescence. There is no direct population evidence that *Kaji-ichigoside F1* can improve arsenic-induced skin cell senescence. Therefore, it is very necessary to conduct more in-depth research (animal experiments and clinical trials) on the biological significance of *Kaji-ichigoside F1* in the treatment of arsenic-induced skin cell senescence. More importantly, in addition to exploring the therapeutic effects of *Kaji-ichigoside F1*, we need to explore the preventive and antagonistic effects of *Kaji-ichigoside F1* in the next step to better understand the role of *Kaji-ichigoside F1* in arsenic-induced skin cell senescence.

## 5. Conclusions

**Figure 4:**
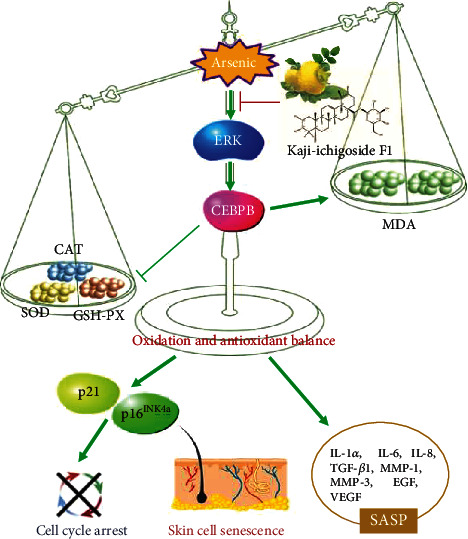
Assessing the potential value and mechanism of *Kaji-ichigoside F1* on arsenite-induced skin cell senescence. Overall, our study provides some limited evidence that the ERK/CEBPB signaling pathway is involved in low-dose arsenic-induced skin cell senescence, through regulating oxidative stress. The second major finding was that *Kaji-ichigoside F1* can downregulate the ERK/CEBPB signaling pathway and regulate the balance between oxidation and antioxidation, alleviating arsenic-induced skin cell senescence.

Overall, our study provides some limited evidence that the ERK/CEBPB signaling pathway is involved in low-dose arsenic-induced skin cell senescence, through regulating oxidative stress (Figure 4). The second major finding was that *Kaji-ichigoside F1* can downregulate the ERK/CEBPB signaling pathway and regulate the balance between oxidation and antioxidation, alleviating arsenic-induced skin cell senescence (Figure 4). This study is the first to comprehensively assess the value and mechanism of *Kaji-ichigoside F1* in the treatment of skin cell senescence. This study provides experimental evidence for further understanding of *Kaji-ichigoside F1*, a natural medicinal plant that may be more effective in preventing and controlling arsenic poisoning.

## Data Availability

The data supporting the conclusions of the study have been uploaded in the supplementary materials.

## Supplementary Materials

Supplementary Materials Supplementary table.pdf: the primer sequences. 2. Data.pdf: the data supporting the conclusions of the study.
