# Supplementary material for: Assessing the Potential Value and Mechanism of Kaji-Ichigoside F1 on Arsenite-Induced Skin Cell Senescence
Source: Oxid Med Cell Longev. 2022 Jan 11;2022:9574473. doi: 10.1155/2022/9574473 (PMC8767413; doi:10.1155/2022/9574473)
Supplement: Supplementary Materials — Supplementary table.pdf: the primer sequences. 2. Data.pdf: the data supporting the conclusions of the study. [file 9574473.f1.zip › Data.pdf]

Group	Time（h）	$NaAsO_2$(μM)	IL-1α(pg/mL)	IL-6(pg/mL)	IL-8(pg/mL)
Exposure	24	0	22.35	43.01	22.36
Exposure	24	0	20.68	37.73	23.98
Exposure	24	0	24.97	45.47	22.75
Exposure	24	0	25.00	49.70	20.54
Exposure	24	0	21.55	42.75	22.34
Exposure	24	0	23.48	44.56	21.43
Exposure	24	0.05	25.42	48.05	26.03
Exposure	24	0.05	27.18	49.49	28.15
Exposure	24	0.05	29.85	57.15	26.47
Exposure	24	0.05	25.19	46.02	25.42
Exposure	24	0.05	25.05	48.12	25.53
Exposure	24	0.05	27.41	50.37	25.83
Exposure	24	0.1	33.99	62.97	36.50
Exposure	24	0.1	30.66	56.46	36.80
Exposure	24	0.1	34.41	66.42	37.64
Exposure	24	0.1	32.82	64.38	36.24
Exposure	24	0.1	30.65	57.21	35.70
Exposure	24	0.1	36.91	70.52	38.18
Exposure	24	0.25	45.97	89.62	54.66
Exposure	24	0.25	42.24	81.01	54.15
Exposure	24	0.25	43.57	83.64	54.82
Exposure	24	0.25	49.26	97.20	56.90
Exposure	24	0.25	47.80	94.09	58.74
Exposure	24	0.25	46.39	92.43	52.81
Exposure	48	0	21.25	40.29	22.03
Exposure	48	0	20.17	36.17	24.96
Exposure	48	0	24.65	49.18	20.13
Exposure	48	0	21.53	41.90	23.99
Exposure	48	0	20.35	39.88	23.13
Exposure	48	0	21.68	40.98	24.59
Exposure	48	0.05	30.26	58.67	31.17
Exposure	48	0.05	29.54	57.81	30.18
Exposure	48	0.05	30.04	58.76	30.36
Exposure	48	0.05	29.81	56.38	31.36
Exposure	48	0.05	30.26	57.74	31.89
Exposure	48	0.05	31.38	61.49	32.71
Exposure	48	0.1	40.80	78.76	41.45
Exposure	48	0.1	43.00	82.05	38.76
Exposure	48	0.1	42.14	79.47	42.36
Exposure	48	0.1	44.73	87.07	39.19
Exposure	48	0.1	42.87	82.02	40.64
Exposure	48	0.1	40.36	79.45	38.47
Exposure	48	0.25	50.61	101.19	58.84
Exposure	48	0.25	53.73	103.64	55.48
Exposure	48	0.25	54.34	106.32	55.90
Exposure	48	0.25	50.49	96.54	59.38
Exposure	48	0.25	51.86	103.71	59.80
Exposure	48	0.25	54.47	108.44	58.25
Exposure	72	0	20.44	38.73	24.45
Exposure	72	0	21.67	42.41	21.63
Exposure	72	0	21.43	41.37	23.93
Exposure	72	0	21.51	41.05	24.61
Exposure	72	0	21.56	39.99	20.01
Exposure	72	0	23.08	43.73	23.44
Exposure	72	0.05	32.46	62.86	31.76
Exposure	72	0.05	29.18	57.79	34.80
Exposure	72	0.05	29.98	59.58	34.75

Group	Time（h）	$NaAsO_2$(μM)	IL-1α(pg/mL)	IL-6(pg/mL)	IL-8(pg/mL)
Exposure	72	0.05	33.57	62.31	33.05
Exposure	72	0.05	30.66	58.69	35.08
Exposure	72	0.05	30.76	56.74	31.65
Exposure	72	0.1	47.90	93.74	40.20
Exposure	72	0.1	47.79	95.45	44.40
Exposure	72	0.1	43.82	87.25	43.78
Exposure	72	0.1	45.30	89.43	40.60
Exposure	72	0.1	44.78	87.56	40.19
Exposure	72	0.1	44.33	84.50	44.12
Exposure	72	0.25	51.94	100.31	63.31
Exposure	72	0.25	50.02	99.72	62.98
Exposure	72	0.25	50.48	96.34	64.09
Exposure	72	0.25	54.29	106.23	62.25
Exposure	72	0.25	52.06	101.85	60.37
Exposure	72	0.25	54.25	108.00	64.60
Intervention	24	0	22.45	41.33	25.05
Intervention	24	0	21.91	42.93	22.21
Intervention	24	0	22.61	43.18	25.28
Intervention	24	0	23.77	42.44	27.03
Intervention	24	0	24.43	40.68	20.82
Intervention	24	0	25.08	46.43	26.06
Intervention	24	0.25	54.18	100.34	65.30
Intervention	24	0.25	52.11	100.52	65.46
Intervention	24	0.25	51.31	98.69	66.82
Intervention	24	0.25	54.80	109.17	62.85
Intervention	24	0.25	54.60	102.07	60.97
Intervention	24	0.25	55.75	108.64	65.35
Intervention	24	*Kaji-ichigoside F1*	15.07	35.16	18.74
Intervention	24	*Kaji-ichigoside F1*	18.30	38.41	22.33
Intervention	24	*Kaji-ichigoside F1*	20.15	38.65	18.14
Intervention	24	*Kaji-ichigoside F1*	20.69	43.71	21.20
Intervention	24	*Kaji-ichigoside F1*	23.64	38.94	22.42
Intervention	24	*Kaji-ichigoside F1*	17.41	43.89	19.52
Intervention	24	0.25+Kaji-ichigoside F1	38.51	78.10	39.25
Intervention	24	0.25+Kaji-ichigoside F1	36.57	77.16	35.85
Intervention	24	0.25+Kaji-ichigoside F1	31.42	72.27	37.40
Intervention	24	0.25+Kaji-ichigoside F1	38.17	75.46	39.70
Intervention	24	0.25+Kaji-ichigoside F1	33.65	79.27	36.39
Intervention	24	0.25+Kaji-ichigoside F1	36.15	75.95	39.78

Group	Time（h）	$NaAsO_2$(μM)	TGF-β1(ng/mL)	MMP1(pg/mL)
Exposure	24	0	6.72	477.97
Exposure	24	0	8.19	407.87
Exposure	24	0	6.77	494.49
Exposure	24	0	7.10	537.84
Exposure	24	0	7.87	466.80
Exposure	24	0	6.83	492.07
Exposure	24	0.05	9.17	525.88
Exposure	24	0.05	8.76	527.90
Exposure	24	0.05	6.75	601.56
Exposure	24	0.05	5.40	509.75
Exposure	24	0.05	7.51	517.77
Exposure	24	0.05	7.94	540.68
Exposure	24	0.1	6.87	676.84
Exposure	24	0.1	10.27	609.24
Exposure	24	0.1	7.27	697.21
Exposure	24	0.1	10.16	686.69
Exposure	24	0.1	9.69	616.49
Exposure	24	0.1	10.36	752.27
Exposure	24	0.25	13.92	942.22
Exposure	24	0.25	12.82	843.46
Exposure	24	0.25	10.16	869.52
Exposure	24	0.25	13.43	1007.19
Exposure	24	0.25	10.42	975.84
Exposure	24	0.25	9.82	962.24
Exposure	48	0	7.91	434.41
Exposure	48	0	5.11	405.13
Exposure	48	0	8.14	531.14
Exposure	48	0	5.76	458.16
Exposure	48	0	7.68	438.32
Exposure	48	0	7.93	450.19
Exposure	48	0.05	8.40	624.51
Exposure	48	0.05	6.69	615.78
Exposure	48	0.05	9.86	622.26
Exposure	48	0.05	7.53	608.01
Exposure	48	0.05	9.66	611.99
Exposure	48	0.05	8.89	651.06
Exposure	48	0.1	11.23	821.66
Exposure	48	0.1	13.33	858.82
Exposure	48	0.1	9.28	838.21
Exposure	48	0.1	13.42	919.14
Exposure	48	0.1	12.34	859.02
Exposure	48	0.1	12.73	836.35
Exposure	48	0.25	14.72	1043.47
Exposure	48	0.25	14.35	1069.64
Exposure	48	0.25	13.58	1108.98
Exposure	48	0.25	14.83	1001.17
Exposure	48	0.25	15.33	1067.54
Exposure	48	0.25	13.32	1123.96
Exposure	72	0	8.85	430.16
Exposure	72	0	8.58	464.07
Exposure	72	0	9.16	453.10
Exposure	72	0	5.20	453.82
Exposure	72	0	6.16	433.19
Exposure	72	0	7.12	480.65
Exposure	72	0.05	7.72	675.85
Exposure	72	0.05	7.22	619.52
Exposure	72	0.05	7.70	639.80

Group	Time（h）	$NaAsO_2$(μM)	TGF-β1(ng/mL)	MMP1(pg/mL)
Exposure	72	0.05	7.63	662.85
Exposure	72	0.05	8.27	623.71
Exposure	72	0.05	7.06	611.28
Exposure	72	0.1	13.40	982.55
Exposure	72	0.1	13.88	988.39
Exposure	72	0.1	10.19	912.08
Exposure	72	0.1	12.07	942.06
Exposure	72	0.1	12.64	910.01
Exposure	72	0.1	13.21	894.19
Exposure	72	0.25	14.02	1047.48
Exposure	72	0.25	14.30	1035.47
Exposure	72	0.25	12.69	996.88
Exposure	72	0.25	16.02	1104.55
Exposure	72	0.25	15.00	1066.71
Exposure	72	0.25	15.89	1117.01
Intervention	24	0	11.75	430.80
Intervention	24	0	10.67	465.84
Intervention	24	0	10.33	455.89
Intervention	24	0	5.74	456.53
Intervention	24	0	8.63	435.98
Intervention	24	0	7.46	483.45
Intervention	24	0.25	16.79	1048.24
Intervention	24	0.25	16.84	1037.45
Intervention	24	0.25	14.22	998.38
Intervention	24	0.25	17.35	1107.41
Intervention	24	0.25	16.99	1068.02
Intervention	24	0.25	18.27	1117.43
Intervention	24	*Kaji-ichigoside F1*	9.27	436.91
Intervention	24	*Kaji-ichigoside F1*	8.16	384.99
Intervention	24	*Kaji-ichigoside F1*	8.92	429.59
Intervention	24	*Kaji-ichigoside F1*	7.44	392.44
Intervention	24	*Kaji-ichigoside F1*	7.81	374.29
Intervention	24	*Kaji-ichigoside F1*	7.34	386.63
Intervention	24	0.25+Kaji-ichigoside F1	13.56	728.32
Intervention	24	0.25+Kaji-ichigoside F1	14.63	733.62
Intervention	24	0.25+Kaji-ichigoside F1	13.36	771.75
Intervention	24	0.25+Kaji-ichigoside F1	12.61	712.04
Intervention	24	0.25+Kaji-ichigoside F1	12.43	726.87
Intervention	24	0.25+Kaji-ichigoside F1	11.06	725.92

Group	Time（h）	$NaAsO_2$(μM)	MMP3(pg/mL)	EGF(pg/mL)	VEGF(pg/mL)
Exposure	24	0	262.45	11.63	53.61
Exposure	24	0	282.13	9.67	58.55
Exposure	24	0	278.19	10.34	57.48
Exposure	24	0	257.28	7.24	45.74
Exposure	24	0	262.93	7.74	49.57
Exposure	24	0	255.47	7.95	53.62
Exposure	24	0.05	311.78	12.60	61.39
Exposure	24	0.05	330.88	10.18	66.10
Exposure	24	0.05	302.98	7.79	60.55
Exposure	24	0.05	295.84	13.00	64.30
Exposure	24	0.05	290.26	10.10	65.63
Exposure	24	0.05	303.97	12.22	59.92
Exposure	24	0.1	406.72	13.10	93.44
Exposure	24	0.1	400.56	17.10	97.55
Exposure	24	0.1	418.55	15.56	93.55
Exposure	24	0.1	398.56	12.92	95.38
Exposure	24	0.1	386.43	15.02	93.57
Exposure	24	0.1	422.13	12.98	100.89
Exposure	24	0.25	556.89	22.68	148.99
Exposure	24	0.25	560.00	18.13	143.06
Exposure	24	0.25	570.87	20.77	148.77
Exposure	24	0.25	574.32	19.49	151.79
Exposure	24	0.25	600.69	23.86	160.55
Exposure	24	0.25	551.58	22.93	145.87
Exposure	48	0	273.94	6.76	50.07
Exposure	48	0	295.93	10.84	63.52
Exposure	48	0	243.84	7.71	41.68
Exposure	48	0	281.05	8.65	52.08
Exposure	48	0	271.18	11.10	57.52
Exposure	48	0	299.23	8.03	54.81
Exposure	48	0.05	343.14	10.03	77.88
Exposure	48	0.05	337.67	10.98	75.04
Exposure	48	0.05	350.68	13.69	71.27
Exposure	48	0.05	347.20	11.47	74.58
Exposure	48	0.05	352.26	12.48	80.86
Exposure	48	0.05	356.73	13.69	84.33
Exposure	48	0.1	441.44	17.58	111.69
Exposure	48	0.1	424.40	17.95	104.77
Exposure	48	0.1	457.43	16.46	112.08
Exposure	48	0.1	415.55	13.98	107.05
Exposure	48	0.1	428.14	15.05	109.33
Exposure	48	0.1	406.37	14.21	99.67
Exposure	48	0.25	593.54	24.71	159.29
Exposure	48	0.25	571.32	22.30	148.47
Exposure	48	0.25	569.09	23.01	156.40
Exposure	48	0.25	604.13	25.02	162.09
Exposure	48	0.25	611.41	21.89	161.28
Exposure	48	0.25	597.49	23.65	158.26
Exposure	72	0	287.65	11.41	61.18
Exposure	72	0	267.79	7.63	50.80
Exposure	72	0	280.46	8.67	59.45
Exposure	72	0	290.90	10.99	55.52
Exposure	72	0	248.64	10.18	45.11
Exposure	72	0	277.61	9.52	53.81
Exposure	72	0.05	347.36	11.49	78.23
Exposure	72	0.05	386.89	14.15	89.02
Exposure	72	0.05	376.40	11.04	86.12

Group	Time（h）	$NaAsO_2$(μM)	MMP3(pg/mL)	EGF(pg/mL)	VEGF(pg/mL)
Exposure	72	0.05	370.92	11.29	80.07
Exposure	72	0.05	393.66	14.50	89.12
Exposure	72	0.05	357.57	13.74	76.81
Exposure	72	0.1	424.72	14.07	100.99
Exposure	72	0.1	462.31	15.72	116.92
Exposure	72	0.1	454.98	17.56	114.40
Exposure	72	0.1	442.45	16.51	105.61
Exposure	72	0.1	435.63	15.21	110.29
Exposure	72	0.1	460.61	16.54	121.09
Exposure	72	0.25	645.68	25.20	172.28
Exposure	72	0.25	627.40	23.62	171.11
Exposure	72	0.25	653.10	24.86	180.56
Exposure	72	0.25	625.80	23.39	172.54
Exposure	72	0.25	603.72	20.92	163.51
Exposure	72	0.25	643.34	23.94	183.65
Intervention	24	0	290.09	13.36	63.61
Intervention	24	0	270.66	9.06	51.72
Intervention	24	0	281.27	10.84	60.50
Intervention	24	0	292.54	12.87	56.92
Intervention	24	0	250.04	11.25	46.42
Intervention	24	0	278.47	12.48	55.36
Intervention	24	0.25	646.18	26.75	173.14
Intervention	24	0.25	627.71	23.80	171.48
Intervention	24	0.25	654.58	25.26	181.47
Intervention	24	0.25	626.80	23.67	174.66
Intervention	24	0.25	603.85	22.97	164.08
Intervention	24	0.25	645.97	26.06	185.28
Intervention	24	*Kaji-ichigoside F1*	258.91	11.79	51.28
Intervention	24	*Kaji-ichigoside F1*	229.83	8.89	41.29
Intervention	24	*Kaji-ichigoside F1*	266.14	10.20	55.96
Intervention	24	*Kaji-ichigoside F1*	288.67	9.37	52.82
Intervention	24	*Kaji-ichigoside F1*	240.79	7.42	44.82
Intervention	24	*Kaji-ichigoside F1*	233.22	9.47	55.61
Intervention	24	0.25+Kaji-ichigoside F1	479.27	21.90	103.60
Intervention	24	0.25+Kaji-ichigoside F1	432.09	18.34	113.61
Intervention	24	0.25+Kaji-ichigoside F1	431.81	19.26	110.63
Intervention	24	0.25+Kaji-ichigoside F1	437.00	19.18	115.62
Intervention	24	0.25+Kaji-ichigoside F1	421.79	21.23	110.86
Intervention	24	0.25+Kaji-ichigoside F1	476.04	18.22	109.52

Group	Time（h）	$NaAsO_2$(μM)	SOD(U/mL prot)	GSH-Px(U/mL prot)
Exposure	24	0	20.06	113.79
Exposure	24	0	16.16	114.47
Exposure	24	0	18.32	96.28
Exposure	24	0	24.34	106.54
Exposure	24	0	22.09	97.03
Exposure	24	0	16.49	106.68
Exposure	24	0.05	13.09	99.99
Exposure	24	0.05	20.23	105.85
Exposure	24	0.05	15.21	100.14
Exposure	24	0.05	14.50	96.96
Exposure	24	0.05	18.73	91.68
Exposure	24	0.05	20.32	109.34
Exposure	24	0.1	14.96	93.17
Exposure	24	0.1	10.03	91.14
Exposure	24	0.1	11.93	93.61
Exposure	24	0.1	14.99	97.21
Exposure	24	0.1	14.28	102.85
Exposure	24	0.1	11.60	98.27
Exposure	24	0.25	12.86	88.34
Exposure	24	0.25	11.68	99.14
Exposure	24	0.25	8.00	89.86
Exposure	24	0.25	12.17	85.28
Exposure	24	0.25	10.98	95.36
Exposure	24	0.25	13.55	88.95
Exposure	48	0	23.09	107.40
Exposure	48	0	16.51	104.97
Exposure	48	0	19.15	96.41
Exposure	48	0	17.53	110.30
Exposure	48	0	23.48	112.08
Exposure	48	0	17.31	102.29
Exposure	48	0.05	14.12	103.79
Exposure	48	0.05	18.63	92.97
Exposure	48	0.05	19.38	95.49
Exposure	48	0.05	12.50	103.96
Exposure	48	0.05	17.33	101.90
Exposure	48	0.05	14.40	93.82
Exposure	48	0.1	13.68	87.73
Exposure	48	0.1	11.66	101.09
Exposure	48	0.1	15.55	90.75
Exposure	48	0.1	19.05	101.21
Exposure	48	0.1	11.98	95.65
Exposure	48	0.1	10.88	92.46
Exposure	48	0.25	10.40	96.63
Exposure	48	0.25	13.91	97.81
Exposure	48	0.25	10.25	91.72
Exposure	48	0.25	10.37	80.58
Exposure	48	0.25	10.26	83.47
Exposure	48	0.25	10.94	92.66
Exposure	72	0	16.51	107.44
Exposure	72	0	18.21	110.53
Exposure	72	0	19.66	108.86
Exposure	72	0	16.71	95.10
Exposure	72	0	13.10	110.39
Exposure	72	0	21.07	99.33
Exposure	72	0.05	12.12	104.56
Exposure	72	0.05	12.26	85.14
Exposure	72	0.05	18.53	100.37

Group	Time（h）	$NaAsO_2$(μM)	SOD(U/mL prot)	GSH-Px(U/mL prot)
Exposure	72	0.05	17.74	90.93
Exposure	72	0.05	16.79	99.07
Exposure	72	0.05	12.33	99.39
Exposure	72	0.1	13.78	96.40
Exposure	72	0.1	15.21	99.07
Exposure	72	0.1	14.40	85.25
Exposure	72	0.1	8.68	91.20
Exposure	72	0.1	8.48	88.65
Exposure	72	0.1	11.51	93.94
Exposure	72	0.25	10.54	97.43
Exposure	72	0.25	9.62	85.73
Exposure	72	0.25	7.41	98.01
Exposure	72	0.25	10.08	88.50
Exposure	72	0.25	15.52	88.44
Exposure	72	0.25	8.99	84.13
Intervention	24	0	21.14	111.02
Intervention	24	0	17.92	110.39
Intervention	24	0	20.29	98.04
Intervention	24	0	21.55	99.60
Intervention	24	0	18.22	106.16
Intervention	24	0	15.18	108.47
Intervention	24	0.25	9.99	97.49
Intervention	24	0.25	15.37	98.40
Intervention	24	0.25	8.59	83.96
Intervention	24	0.25	14.95	83.39
Intervention	24	0.25	10.49	83.65
Intervention	24	0.25	6.87	96.66
Intervention	24	*Kaji-ichigoside F1*	25.43	117.32
Intervention	24	*Kaji-ichigoside F1*	18.14	119.03
Intervention	24	*Kaji-ichigoside F1*	18.15	125.34
Intervention	24	*Kaji-ichigoside F1*	17.30	126.46
Intervention	24	*Kaji-ichigoside F1*	16.61	129.97
Intervention	24	*Kaji-ichigoside F1*	20.32	127.71
Intervention	24	0.25+Kaji-ichigoside F1	17.47	107.92
Intervention	24	0.25+Kaji-ichigoside F1	12.74	97.44
Intervention	24	0.25+Kaji-ichigoside F1	15.12	102.13
Intervention	24	0.25+Kaji-ichigoside F1	11.22	92.10
Intervention	24	0.25+Kaji-ichigoside F1	14.83	108.73
Intervention	24	0.25+Kaji-ichigoside F1	18.50	93.21

Group	Time（h）	$NaAsO_2$(μM)	GST(U/mL prot)	MDA(mmol/mL prot)
Exposure	24	0	24.85	4.78
Exposure	24	0	25.62	4.25
Exposure	24	0	26.58	3.96
Exposure	24	0	28.06	3.39
Exposure	24	0	25.61	3.62
Exposure	24	0	24.30	3.62
Exposure	24	0.05	21.71	3.83
Exposure	24	0.05	22.04	3.71
Exposure	24	0.05	25.94	4.63
Exposure	24	0.05	23.42	3.53
Exposure	24	0.05	24.41	4.88
Exposure	24	0.05	25.81	4.76
Exposure	24	0.1	20.88	4.89
Exposure	24	0.1	24.12	4.88
Exposure	24	0.1	20.80	3.76
Exposure	24	0.1	21.84	3.76
Exposure	24	0.1	22.14	5.26
Exposure	24	0.1	23.51	4.91
Exposure	24	0.25	19.50	5.09
Exposure	24	0.25	20.99	4.88
Exposure	24	0.25	20.38	4.59
Exposure	24	0.25	20.30	5.18
Exposure	24	0.25	19.76	4.37
Exposure	24	0.25	20.85	4.72
Exposure	48	0	25.41	3.46
Exposure	48	0	26.92	4.28
Exposure	48	0	23.28	4.77
Exposure	48	0	24.78	3.64
Exposure	48	0	26.10	3.40
Exposure	48	0	24.29	3.86
Exposure	48	0.05	21.03	4.45
Exposure	48	0.05	22.26	4.31
Exposure	48	0.05	23.56	5.06
Exposure	48	0.05	22.09	4.23
Exposure	48	0.05	24.53	4.36
Exposure	48	0.05	24.49	4.32
Exposure	48	0.1	20.30	4.30
Exposure	48	0.1	21.81	5.20
Exposure	48	0.1	22.90	4.20
Exposure	48	0.1	20.94	5.11
Exposure	48	0.1	20.90	4.73
Exposure	48	0.1	22.27	5.50
Exposure	48	0.25	18.30	5.47
Exposure	48	0.25	18.65	4.95
Exposure	48	0.25	17.14	4.83
Exposure	48	0.25	20.04	5.69
Exposure	48	0.25	17.92	4.13
Exposure	48	0.25	21.98	5.37
Exposure	72	0	26.82	3.26
Exposure	72	0	23.43	4.78
Exposure	72	0	26.05	4.47
Exposure	72	0	25.80	3.16
Exposure	72	0	26.41	3.33
Exposure	72	0	24.35	4.44
Exposure	72	0.05	23.64	4.48
Exposure	72	0.05	23.66	5.21
Exposure	72	0.05	21.67	4.72

Group	Time（h）	$NaAsO_2$(μM)	GST(U/mL prot)	MDA(mmol/mL prot)
Exposure	72	0.05	21.89	3.53
Exposure	72	0.05	20.79	4.90
Exposure	72	0.05	20.86	4.94
Exposure	72	0.1	19.41	4.68
Exposure	72	0.1	19.76	4.01
Exposure	72	0.1	22.35	4.77
Exposure	72	0.1	22.20	4.03
Exposure	72	0.1	21.62	3.41
Exposure	72	0.1	18.55	3.53
Exposure	72	0.25	16.47	5.09
Exposure	72	0.25	18.12	5.67
Exposure	72	0.25	20.92	4.28
Exposure	72	0.25	20.95	4.44
Exposure	72	0.25	17.95	5.83
Exposure	72	0.25	16.06	5.42
Intervention	24	0	27.77	3.19
Intervention	24	0	24.73	3.75
Intervention	24	0	23.39	3.34
Intervention	24	0	27.88	3.54
Intervention	24	0	23.32	4.67
Intervention	24	0	26.91	4.98
Intervention	24	0.25	16.98	4.62
Intervention	24	0.25	16.89	5.76
Intervention	24	0.25	17.29	5.04
Intervention	24	0.25	17.63	4.50
Intervention	24	0.25	18.83	4.95
Intervention	24	0.25	20.70	6.03
Intervention	24	*Kaji-ichigoside F1*	28.83	3.82
Intervention	24	*Kaji-ichigoside F1*	26.27	2.50
Intervention	24	*Kaji-ichigoside F1*	29.80	3.25
Intervention	24	*Kaji-ichigoside F1*	28.09	4.14
Intervention	24	*Kaji-ichigoside F1*	29.72	2.43
Intervention	24	*Kaji-ichigoside F1*	28.44	2.45
Intervention	24	0.25+Kaji-ichigoside F1	24.71	4.41
Intervention	24	0.25+Kaji-ichigoside F1	21.29	4.37
Intervention	24	0.25+Kaji-ichigoside F1	24.86	5.12
Intervention	24	0.25+Kaji-ichigoside F1	23.16	4.55
Intervention	24	0.25+Kaji-ichigoside F1	21.81	3.55
Intervention	24	0.25+Kaji-ichigoside F1	20.97	4.75

Group	Time（h）	$NaAsO_2$(μM)	mRNA ERK1	mRNA ERK2	mRNA CEBPB
Exposure	24	0	0.947	0.982	0.971
Exposure	24	0	1.024	0.989	1.020
Exposure	24	0	0.988	0.982	0.964
Exposure	24	0	0.977	1.004	0.988
Exposure	24	0	1.041	1.019	1.058
Exposure	24	0	1.025	1.024	1.002
Exposure	24	0.05	1.159	1.146	1.240
Exposure	24	0.05	1.120	1.069	1.064
Exposure	24	0.05	1.067	1.146	1.320
Exposure	24	0.05	1.177	1.165	1.264
Exposure	24	0.05	1.161	1.081	1.106
Exposure	24	0.05	1.101	1.189	1.346
Exposure	24	0.1	1.313	1.289	1.257
Exposure	24	0.1	1.313	1.186	1.197
Exposure	24	0.1	1.184	1.245	1.266
Exposure	24	0.1	1.337	1.324	1.299
Exposure	24	0.1	1.333	1.229	1.244
Exposure	24	0.1	1.217	1.268	1.296
Exposure	24	0.25	1.517	1.545	1.774
Exposure	24	0.25	1.595	1.486	1.583
Exposure	24	0.25	1.451	1.369	1.704
Exposure	24	0.25	1.543	1.576	1.790
Exposure	24	0.25	1.616	1.531	1.629
Exposure	24	0.25	1.473	1.401	1.746
Exposure	48	0	0.970	1.059	0.843
Exposure	48	0	0.937	0.940	0.958
Exposure	48	0	1.048	0.974	1.152
Exposure	48	0	1.016	1.075	0.888
Exposure	48	0	0.965	0.963	0.970
Exposure	48	0	1.066	0.994	1.189
Exposure	48	0.05	1.959	1.941	1.332
Exposure	48	0.05	1.853	2.080	1.192
Exposure	48	0.05	1.866	1.941	1.243
Exposure	48	0.05	1.996	1.975	1.357
Exposure	48	0.05	1.865	2.102	1.222
Exposure	48	0.05	1.895	1.980	1.254
Exposure	48	0.1	2.071	2.109	1.807
Exposure	48	0.1	2.056	2.066	1.896
Exposure	48	0.1	2.219	1.968	1.769
Exposure	48	0.1	2.083	2.120	1.851
Exposure	48	0.1	2.096	2.116	1.937
Exposure	48	0.1	2.254	1.983	1.798
Exposure	48	0.25	2.514	2.199	2.119
Exposure	48	0.25	2.329	2.261	2.061
Exposure	48	0.25	2.497	2.308	2.019
Exposure	48	0.25	2.554	2.222	2.152
Exposure	48	0.25	2.359	2.300	2.080
Exposure	48	0.25	2.516	2.354	2.045
Exposure	72	0	1.033	0.967	1.005
Exposure	72	0	0.916	1.023	1.019
Exposure	72	0	1.004	0.946	0.916
Exposure	72	0	1.077	1.009	1.033
Exposure	72	0	0.931	1.067	1.066
Exposure	72	0	1.040	0.988	0.962
Exposure	72	0.05	3.283	3.323	2.977
Exposure	72	0.05	3.125	3.217	3.026
Exposure	72	0.05	3.165	3.243	3.055

Group	Time（h）	$NaAsO_2$(μM)	mRNA ERK1	mRNA ERK2	mRNA CEBPB
Exposure	72	0.05	3.333	3.370	3.013
Exposure	72	0.05	3.171	3.229	3.037
Exposure	72	0.05	3.213	3.269	3.092
Exposure	72	0.1	3.338	3.379	3.130
Exposure	72	0.1	3.165	3.379	3.162
Exposure	72	0.1	3.292	3.457	3.122
Exposure	72	0.1	3.385	3.401	3.144
Exposure	72	0.1	3.206	3.407	3.173
Exposure	72	0.1	3.311	3.479	3.162
Exposure	72	0.25	3.495	3.662	3.211
Exposure	72	0.25	3.329	3.721	3.289
Exposure	72	0.25	3.301	3.674	3.170
Exposure	72	0.25	3.515	3.693	3.258
Exposure	72	0.25	3.361	3.751	3.306
Exposure	72	0.25	3.315	3.691	3.206
Intervention	24	0	1.005	0.991	0.993
Intervention	24	0	1.012	0.917	1.029
Intervention	24	0	0.943	1.041	0.919
Intervention	24	0	1.042	1.028	1.040
Intervention	24	0	1.028	0.957	1.075
Intervention	24	0	0.970	1.066	0.946
Intervention	24	0.25	3.220	3.200	3.173
Intervention	24	0.25	3.162	3.225	3.248
Intervention	24	0.25	3.146	3.217	3.094
Intervention	24	0.25	3.251	3.233	3.195
Intervention	24	0.25	3.198	3.270	3.279
Intervention	24	0.25	3.192	3.263	3.132
Intervention	24	*Kaji-ichigoside F1*	0.922	0.864	0.886
Intervention	24	*Kaji-ichigoside F1*	0.901	0.864	0.895
Intervention	24	*Kaji-ichigoside F1*	0.817	0.841	0.895
Intervention	24	*Kaji-ichigoside F1*	0.968	0.903	0.899
Intervention	24	*Kaji-ichigoside F1*	0.951	0.897	0.933
Intervention	24	*Kaji-ichigoside F1*	0.845	0.875	0.915
Intervention	24	0.25+Kaji-ichigoside F1	1.881	1.749	1.959
Intervention	24	0.25+Kaji-ichigoside F1	1.905	1.903	1.847
Intervention	24	0.25+Kaji-ichigoside F1	1.875	1.861	1.807
Intervention	24	0.25+Kaji-ichigoside F1	1.908	1.797	1.971
Intervention	24	0.25+Kaji-ichigoside F1	1.937	1.922	1.887
Intervention	24	0.25+Kaji-ichigoside F1	1.888	1.893	1.822

Group	Time（h）	$NaAsO_2$(μM)	mRNA P21	mRNA P16	Protein ERK1
Exposure	24	0	0.977	1.000	1.017
Exposure	24	0	0.985	1.054	0.998
Exposure	24	0	1.009	1.006	1.011
Exposure	24	0	1.046	0.973	1.011
Exposure	24	0	0.976	1.033	0.964
Exposure	24	0	1.011	0.962	0.999
Exposure	24	0.05	1.271	1.213	1.373
Exposure	24	0.05	1.291	1.192	1.489
Exposure	24	0.05	1.260	1.178	1.482
Exposure	24	0.05	1.279	1.215	1.549
Exposure	24	0.05	1.282	1.172	1.585
Exposure	24	0.05	1.277	1.185	1.382
Exposure	24	0.1	1.413	1.506	2.030
Exposure	24	0.1	1.375	1.515	1.827
Exposure	24	0.1	1.404	1.514	1.372
Exposure	24	0.1	1.346	1.489	1.441
Exposure	24	0.1	1.395	1.468	2.090
Exposure	24	0.1	1.349	1.507	1.520
Exposure	24	0.25	1.942	1.942	1.205
Exposure	24	0.25	2.008	2.008	2.229
Exposure	24	0.25	1.998	1.998	1.787
Exposure	24	0.25	2.032	2.032	1.964
Exposure	24	0.25	2.004	2.004	1.680
Exposure	24	0.25	2.000	2.000	2.330
Exposure	48	0	0.960	1.016	0.979
Exposure	48	0	1.026	0.991	1.012
Exposure	48	0	0.999	0.978	1.045
Exposure	48	0	1.013	0.997	0.954
Exposure	48	0	0.989	0.990	1.024
Exposure	48	0	1.024	1.032	0.987
Exposure	48	0.05	1.385	1.384	2.221
Exposure	48	0.05	1.356	1.348	1.411
Exposure	48	0.05	1.425	1.434	1.326
Exposure	48	0.05	1.331	1.422	1.647
Exposure	48	0.05	1.375	1.376	1.003
Exposure	48	0.05	1.386	1.377	1.561
Exposure	48	0.1	1.647	1.502	1.646
Exposure	48	0.1	1.610	1.448	1.875
Exposure	48	0.1	1.620	1.470	2.126
Exposure	48	0.1	1.584	1.519	1.370
Exposure	48	0.1	1.548	1.500	1.697
Exposure	48	0.1	1.592	1.529	1.973
Exposure	48	0.25	2.499	2.591	2.481
Exposure	48	0.25	2.523	2.613	2.854
Exposure	48	0.25	2.509	2.567	2.278
Exposure	48	0.25	2.481	2.616	2.435
Exposure	48	0.25	2.507	2.585	2.402
Exposure	48	0.25	0.972	0.984	2.724
Exposure	72	0	0.990	1.033	0.969
Exposure	72	0	0.963	0.994	1.011
Exposure	72	0	1.023	0.990	1.005
Exposure	72	0	1.027	0.983	1.008
Exposure	72	0	1.030	1.017	0.998
Exposure	72	0	1.586	1.551	1.009
Exposure	72	0.05	1.537	1.551	0.744
Exposure	72	0.05	1.542	1.544	1.091
Exposure	72	0.05	1.510	1.535	1.049

Group	Time（h）	$NaAsO_2$(μM)	mRNA P21	mRNA P16	Protein ERK1
Exposure	72	0.05	1.523	1.560	1.065
Exposure	72	0.05	1.614	1.556	1.113
Exposure	72	0.05	1.740	1.823	1.091
Exposure	72	0.1	1.701	1.811	1.225
Exposure	72	0.1	1.694	1.841	1.084
Exposure	72	0.1	1.678	1.734	1.284
Exposure	72	0.1	1.682	1.778	1.411
Exposure	72	0.1	1.711	1.814	1.200
Exposure	72	0.1	2.502	2.582	1.254
Exposure	72	0.25	2.423	2.574	1.891
Exposure	72	0.25	2.494	2.613	1.644
Exposure	72	0.25	2.500	2.653	2.042
Exposure	72	0.25	2.500	2.606	1.746
Exposure	72	0.25	2.486	2.615	1.917
Exposure	72	0.25	2.427	2.576	1.868
Intervention	24	0	0.950	1.011	0.991
Intervention	24	0	1.041	0.951	1.034
Intervention	24	0	0.990	1.017	1.036
Intervention	24	0	0.978	0.991	0.971
Intervention	24	0	1.017	1.059	0.950
Intervention	24	0	1.032	1.001	1.018
Intervention	24	0.25	2.493	2.515	2.079
Intervention	24	0.25	2.483	2.497	1.407
Intervention	24	0.25	2.525	2.554	1.590
Intervention	24	0.25	2.452	2.504	1.572
Intervention	24	0.25	2.529	2.504	1.859
Intervention	24	0.25	2.510	2.593	1.317
Intervention	24	*Kaji-ichigoside F1*	0.854	0.902	1.125
Intervention	24	*Kaji-ichigoside F1*	0.883	0.881	0.936
Intervention	24	*Kaji-ichigoside F1*	0.841	0.916	1.022
Intervention	24	*Kaji-ichigoside F1*	0.845	0.927	1.126
Intervention	24	*Kaji-ichigoside F1*	0.868	0.886	0.735
Intervention	24	*Kaji-ichigoside F1*	0.917	0.946	0.689
Intervention	24	0.25+Kaji-ichigoside F1	1.480	1.728	1.113
Intervention	24	0.25+Kaji-ichigoside F1	1.489	1.694	0.949
Intervention	24	0.25+Kaji-ichigoside F1	1.519	1.723	1.437
Intervention	24	0.25+Kaji-ichigoside F1	1.575	1.663	1.300
Intervention	24	0.25+Kaji-ichigoside F1	1.575	1.663	1.317
Intervention	24	0.25+Kaji-ichigoside F1	1.475	1.463	1.150

Group	Time（h）	$NaAsO_2$(μM)	Protein ERK2	Protein p-ERK
Exposure	24	0	0.984	0.996
Exposure	24	0	0.977	0.962
Exposure	24	0	1.033	1.006
Exposure	24	0	1.001	1.016
Exposure	24	0	1.012	1.011
Exposure	24	0	0.994	1.009
Exposure	24	0.05	2.284	4.005
Exposure	24	0.05	2.007	3.752
Exposure	24	0.05	2.044	5.442
Exposure	24	0.05	1.988	3.439
Exposure	24	0.05	1.943	4.082
Exposure	24	0.05	1.677	4.303
Exposure	24	0.1	2.845	4.574
Exposure	24	0.1	2.577	2.886
Exposure	24	0.1	1.468	4.373
Exposure	24	0.1	1.853	3.276
Exposure	24	0.1	2.769	4.149
Exposure	24	0.1	3.175	3.870
Exposure	24	0.25	3.319	2.188
Exposure	24	0.25	2.294	2.094
Exposure	24	0.25	3.309	2.174
Exposure	24	0.25	3.043	1.653
Exposure	24	0.25	2.623	1.652
Exposure	24	0.25	2.334	1.887
Exposure	48	0	1.022	1.009
Exposure	48	0	0.983	0.997
Exposure	48	0	1.011	0.983
Exposure	48	0	1.000	1.020
Exposure	48	0	1.049	0.987
Exposure	48	0	0.935	1.004
Exposure	48	0.05	1.591	1.609
Exposure	48	0.05	2.053	1.734
Exposure	48	0.05	1.756	1.552
Exposure	48	0.05	1.874	1.113
Exposure	48	0.05	1.314	1.269
Exposure	48	0.05	1.454	1.374
Exposure	48	0.1	1.117	1.667
Exposure	48	0.1	1.334	1.096
Exposure	48	0.1	1.676	1.429
Exposure	48	0.1	1.911	1.361
Exposure	48	0.1	1.650	1.747
Exposure	48	0.1	3.222	1.220
Exposure	48	0.25	2.725	0.990
Exposure	48	0.25	3.568	1.065
Exposure	48	0.25	4.018	1.574
Exposure	48	0.25	2.255	1.837
Exposure	48	0.25	2.014	1.519
Exposure	48	0.25	3.009	1.748
Exposure	72	0	1.048	1.017
Exposure	72	0	1.042	0.975
Exposure	72	0	0.978	1.007
Exposure	72	0	1.035	1.051
Exposure	72	0	0.918	1.005
Exposure	72	0	0.979	0.946
Exposure	72	0.05	1.572	1.811
Exposure	72	0.05	1.622	1.873
Exposure	72	0.05	1.477	2.139

Group	Time（h)	$NaAsO_2$(μM)	Protein ERK2	Protein p-ERK
Exposure	72	0.05	1.081	2.223
Exposure	72	0.05	1.019	1.733
Exposure	72	0.05	0.915	1.998
Exposure	72	0.1	1.772	1.148
Exposure	72	0.1	1.955	2.331
Exposure	72	0.1	1.582	1.875
Exposure	72	0.1	1.254	2.481
Exposure	72	0.1	1.500	1.391
Exposure	72	0.1	1.238	2.130
Exposure	72	0.25	2.673	2.802
Exposure	72	0.25	3.035	2.379
Exposure	72	0.25	1.959	3.142
Exposure	72	0.25	2.734	3.096
Exposure	72	0.25	2.702	2.302
Exposure	72	0.25	3.457	2.533
Intervention	24	0	1.009	1.006
Intervention	24	0	1.002	0.980
Intervention	24	0	1.025	1.023
Intervention	24	0	0.994	1.010
Intervention	24	0	1.032	0.965
Intervention	24	0	0.938	1.016
Intervention	24	0.25	1.398	1.965
Intervention	24	0.25	1.301	2.034
Intervention	24	0.25	1.933	2.139
Intervention	24	0.25	1.291	2.289
Intervention	24	0.25	2.214	2.115
Intervention	24	0.25	1.541	2.078
Intervention	24	*Kaji-ichigoside F1*	0.510	0.500
Intervention	24	*Kaji-ichigoside F1*	0.672	0.685
Intervention	24	*Kaji-ichigoside F1*	0.633	0.498
Intervention	24	*Kaji-ichigoside F1*	0.646	0.616
Intervention	24	*Kaji-ichigoside F1*	0.742	0.735
Intervention	24	*Kaji-ichigoside F1*	0.748	0.759
Intervention	24	0.25+Kaji-ichigoside F1	0.724	1.135
Intervention	24	0.25+Kaji-ichigoside F1	1.063	0.852
Intervention	24	0.25+Kaji-ichigoside F1	0.975	1.084
Intervention	24	0.25+Kaji-ichigoside F1	0.891	1.063
Intervention	24	0.25+Kaji-ichigoside F1	0.782	0.813
Intervention	24	0.25+Kaji-ichigoside F1	0.869	0.919

Group	Time（h）	$NaAsO_2$(μM)	Protein CEBPB	Protein p21
Exposure	24	0	1.000	0.990
Exposure	24	0	1.025	0.983
Exposure	24	0	1.026	1.006
Exposure	24	0	0.945	1.008
Exposure	24	0	1.003	0.999
Exposure	24	0	1.002	1.013
Exposure	24	0.05	1.977	2.091
Exposure	24	0.05	1.521	1.816
Exposure	24	0.05	1.865	1.838
Exposure	24	0.05	1.268	1.965
Exposure	24	0.05	1.643	2.230
Exposure	24	0.05	1.889	2.723
Exposure	24	0.1	2.893	5.085
Exposure	24	0.1	2.578	3.465
Exposure	24	0.1	2.703	3.976
Exposure	24	0.1	3.152	4.558
Exposure	24	0.1	2.472	6.218
Exposure	24	0.1	3.389	4.107
Exposure	24	0.25	5.183	6.848
Exposure	24	0.25	3.805	8.480
Exposure	24	0.25	7.013	7.726
Exposure	24	0.25	3.385	4.060
Exposure	24	0.25	8.201	7.779
Exposure	24	0.25	5.173	7.239
Exposure	48	0	0.983	0.990
Exposure	48	0	0.959	0.993
Exposure	48	0	1.034	1.023
Exposure	48	0	0.980	0.990
Exposure	48	0	1.030	1.022
Exposure	48	0	1.013	0.982
Exposure	48	0.05	2.155	1.874
Exposure	48	0.05	1.558	1.278
Exposure	48	0.05	1.350	1.829
Exposure	48	0.05	1.427	2.001
Exposure	48	0.05	1.643	1.489
Exposure	48	0.05	1.273	1.313
Exposure	48	0.1	1.730	1.454
Exposure	48	0.1	1.419	2.016
Exposure	48	0.1	1.439	1.951
Exposure	48	0.1	1.684	2.146
Exposure	48	0.1	1.681	2.055
Exposure	48	0.1	1.629	1.872
Exposure	48	0.25	2.523	1.780
Exposure	48	0.25	1.800	1.967
Exposure	48	0.25	1.756	2.236
Exposure	48	0.25	1.688	1.625
Exposure	48	0.25	2.069	2.208
Exposure	48	0.25	1.963	2.170
Exposure	72	0	0.982	0.969
Exposure	72	0	1.061	1.030
Exposure	72	0	0.963	1.021
Exposure	72	0	0.954	0.981
Exposure	72	0	1.011	1.024
Exposure	72	0	1.028	0.976
Exposure	72	0.05	1.621	1.292
Exposure	72	0.05	1.370	1.177
Exposure	72	0.05	2.022	1.655

Group	Time（h）	$NaAsO_2$(μM)	Protein CEBPB	Protein p21
Exposure	72	0.05	1.285	1.590
Exposure	72	0.05	1.461	1.668
Exposure	72	0.05	1.048	1.395
Exposure	72	0.1	1.297	1.760
Exposure	72	0.1	1.370	2.110
Exposure	72	0.1	1.347	2.144
Exposure	72	0.1	1.267	1.664
Exposure	72	0.1	1.976	2.467
Exposure	72	0.1	1.661	1.903
Exposure	72	0.25	1.333	4.311
Exposure	72	0.25	2.052	3.645
Exposure	72	0.25	0.725	2.855
Exposure	72	0.25	1.795	2.791
Exposure	72	0.25	1.430	2.192
Exposure	72	0.25	1.533	3.536
Intervention	24	0	0.981	1.004
Intervention	24	0	1.059	0.960
Intervention	24	0	0.979	1.044
Intervention	24	0	0.983	0.989
Intervention	24	0	1.009	1.020
Intervention	24	0	0.989	0.983
Intervention	24	0.25	3.140	1.677
Intervention	24	0.25	1.874	1.680
Intervention	24	0.25	2.925	1.416
Intervention	24	0.25	2.111	1.319
Intervention	24	0.25	2.399	1.445
Intervention	24	0.25	2.000	1.211
Intervention	24	*Kaji-ichigoside F1*	1.205	0.820
Intervention	24	*Kaji-ichigoside F1*	1.132	0.703
Intervention	24	*Kaji-ichigoside F1*	1.140	0.618
Intervention	24	*Kaji-ichigoside F1*	1.787	0.529
Intervention	24	*Kaji-ichigoside F1*	1.435	0.843
Intervention	24	*Kaji-ichigoside F1*	0.899	1.071
Intervention	24	0.25+Kaji-ichigoside F1	1.609	1.446
Intervention	24	0.25+Kaji-ichigoside F1	1.702	1.085
Intervention	24	0.25+Kaji-ichigoside F1	1.463	1.275
Intervention	24	0.25+Kaji-ichigoside F1	1.690	1.127
Intervention	24	0.25+Kaji-ichigoside F1	1.269	1.055
Intervention	24	0.25+Kaji-ichigoside F1	1.525	1.119

Group	Time（h）	$NaAsO_2$(μM)	Protein p16	G1	S	G2
Exposure	24	0	1.039	25.4	53.2	16.0
Exposure	24	0	1.000	27.5	54.1	14.7
Exposure	24	0	0.967	28.6	51.0	16.9
Exposure	24	0	0.966	26.8	53.9	15.4
Exposure	24	0	1.039	26.6	51.2	15.1
Exposure	24	0	0.988	26.9	52.9	16.1
Exposure	24	0.05	1.095	29.1	49.5	17.7
Exposure	24	0.05	0.839	29.2	51.6	17.5
Exposure	24	0.05	1.095	30.6	51.6	13.7
Exposure	24	0.05	1.109	30.5	50.7	16.9
Exposure	24	0.05	0.572	29.9	51.0	17.0
Exposure	24	0.05	1.393	29.5	51.2	16.3
Exposure	24	0.1	0.881	34.8	48.0	16.9
Exposure	24	0.1	1.413	34.8	48.7	13.4
Exposure	24	0.1	1.100	34.9	45.4	15.3
Exposure	24	0.1	1.181	34.8	45.4	13.6
Exposure	24	0.1	0.964	34.9	46.6	15.9
Exposure	24	0.1	1.305	34.8	46.9	15.9
Exposure	24	0.25	1.298	36.5	40.6	20.9
Exposure	24	0.25	1.295	37.4	45.4	15.3
Exposure	24	0.25	1.340	37.8	44.1	17.4
Exposure	24	0.25	1.114	37.1	41.2	20.6
Exposure	24	0.25	1.198	37.1	40.6	15.8
Exposure	24	0.25	1.126	36.6	41.3	17.8
Exposure	48	0	1.015	32.9	43.7	19.3
Exposure	48	0	0.994	33.6	43.3	20.9
Exposure	48	0	0.969	37.9	37.3	19.7
Exposure	48	0	1.039	33.9	40.6	20.5
Exposure	48	0	1.022	37.6	38.3	19.4
Exposure	48	0	0.960	35.0	42.6	19.3
Exposure	48	0.05	1.290	38.9	36.7	21.5
Exposure	48	0.05	1.527	39.0	36.2	23.4
Exposure	48	0.05	1.177	39.2	37.3	21.6
Exposure	48	0.05	1.211	39.1	37.3	23.2
Exposure	48	0.05	0.979	39.1	36.9	22.6
Exposure	48	0.05	1.464	38.9	36.9	22.7
Exposure	48	0.1	1.251	45.4	30.9	17.1
Exposure	48	0.1	1.108	45.7	28.1	19.5
Exposure	48	0.1	1.596	49.3	27.5	17.0
Exposure	48	0.1	1.649	46.0	30.7	19.2
Exposure	48	0.1	1.124	47.6	30.9	18.3
Exposure	48	0.1	1.033	46.3	29.4	17.3
Exposure	48	0.25	1.281	50.9	27.4	16.6
Exposure	48	0.25	1.725	53.8	24.8	20.3
Exposure	48	0.25	1.319	56.0	23.7	20.2
Exposure	48	0.25	1.125	55.3	23.8	19.4
Exposure	48	0.25	1.109	55.4	26.4	17.2
Exposure	48	0.25	1.541	51.6	24.2	18.2
Exposure	72	0	1.037	45.9	33.8	15.1
Exposure	72	0	1.048	43.7	38.1	13.9
Exposure	72	0	0.968	45.0	37.4	14.0
Exposure	72	0	1.000	44.1	37.2	14.6
Exposure	72	0	0.979	43.7	34.8	14.8
Exposure	72	0	0.969	44.5	36.7	14.7
Exposure	72	0.05	0.895	46.3	38.0	13.7
Exposure	72	0.05	0.806	47.6	34.2	15.0
Exposure	72	0.05	1.268	47.2	36.1	14.8

Group	Time (h)	$NaAsO_2$(μM)	Protein p16	G1	S	G2
Exposure	72	0.05	1.087	47.5	36.5	15.0
Exposure	72	0.05	0.759	47.5	36.4	14.4
Exposure	72	0.05	1.196	47.1	35.1	17.1
Exposure	72	0.1	1.103	48.3	35.3	13.8
Exposure	72	0.1	1.285	49.8	32.1	15.0
Exposure	72	0.1	1.629	49.6	32.4	14.8
Exposure	72	0.1	1.186	48.8	33.3	14.8
Exposure	72	0.1	1.343	49.0	34.6	14.5
Exposure	72	0.1	1.769	49.4	34.3	14.9
Exposure	72	0.25	2.196	50.0	32.2	14.5
Exposure	72	0.25	1.577	52.3	31.3	15.1
Exposure	72	0.25	1.359	52.6	30.1	15.4
Exposure	72	0.25	1.539	51.6	31.3	15.1
Exposure	72	0.25	1.720	51.7	30.5	14.6
Exposure	72	0.25	1.657	51.1	31.6	15.4
Intervention	24	0	0.993	52.1	27.6	14.5
Intervention	24	0	1.024	52.3	28.9	13.6
Intervention	24	0	1.008	52.8	27.9	14.2
Intervention	24	0	0.997	52.5	28.8	13.6
Intervention	24	0	0.988	52.4	28.6	14.3
Intervention	24	0	0.989	52.2	28.8	13.6
Intervention	24	0.25	1.341	59.0	20.8	16.0
Intervention	24	0.25	0.980	58.6	22.5	15.1
Intervention	24	0.25	1.083	59.9	21.1	15.6
Intervention	24	0.25	1.408	59.8	21.4	15.5
Intervention	24	0.25	1.172	59.2	22.3	15.2
Intervention	24	0.25	1.723	59.8	21.6	15.4
Intervention	24	*Kaji-ichigoside F1*	0.675	45.3	34.3	13.8
Intervention	24	*Kaji-ichigoside F1*	0.817	46.6	35.7	11.8
Intervention	24	*Kaji-ichigoside F1*	0.598	46.8	34.2	13.1
Intervention	24	*Kaji-ichigoside F1*	0.790	46.7	34.6	13.3
Intervention	24	*Kaji-ichigoside F1*	0.833	45.7	34.5	12.9
Intervention	24	*Kaji-ichigoside F1*	0.544	45.9	35.3	13.1
Intervention	24	0.25+Kaji-ichigoside F1	1.285	54.0	26.2	13.5
Intervention	24	0.25+Kaji-ichigoside F1	0.885	54.9	24.5	14.2
Intervention	24	0.25+Kaji-ichigoside F1	1.126	55.5	22.8	15.9
Intervention	24	0.25+Kaji-ichigoside F1	1.017	54.4	25.8	13.7
Intervention	24	0.25+Kaji-ichigoside F1	1.173	54.8	25.7	13.8
Intervention	24	0.25+Kaji-ichigoside F1	1.053	55.1	25.6	15.4
