# Supplementary material for: Assessing the Potential Value and Mechanism of Kaji-Ichigoside F1 on Arsenite-Induced Skin Cell Senescence
Source: Oxid Med Cell Longev. 2022 Jan 11;2022:9574473. doi: 10.1155/2022/9574473 (PMC8767413; doi:10.1155/2022/9574473)
Supplement: Supplementary Materials — Supplementary table.pdf: the primer sequences. 2. Data.pdf: the data supporting the conclusions of the study. [file 9574473.f1.zip › Supplementary table (1).pdf]

Table S1 Primer sequences

Gene	Primer sequences
**ERK1-F**	5'-ATCTGCTACTTCCTCTACC-3'
**ERK1-R**	5'-CCGAAATCACAAATCTTAA-3'
**EKR2-F**	5'-ATTTGGTCTGTAGGCTGC-3'
**ERK2-R**	5'-TCTTCTTGTGATGGGGAT-3'
**CEBPB-F**	5'-TGCGGGGATCTTGAAGCTTA-3'
**CEBPB-R**	5'-GAGCACTCGATGGGGTTAGA-3'
$p21^{INK4a}$**-F**	5'-GACACCACTGGAGGGTGACT-3'
$p21^{INK4a}$**-R**	5'-CAGGTCCACATGGTCTTCCT-3'
***p16*-F**	5'-CCAACGCACCGAATAGTTACG-3'
***p16*-R**	5'-GCGCTGCCCATCATCATG-3'
**GAPDH-F**	5'- GGAAGATGGTGATGGGTTTC -3'
**GAPDH-R**	5'- ATGACTCTACCCACGGCAAG -3'
